# Artificial Intelligence as a Catalyst for Novel Therapeutic Opportunities in Precision Oncology

**DOI:** 10.3390/ijms27198489

**Published:** 2026-09-23

**Authors:** Kostas A. Papavassiliou, Amalia A. Sofianidi, Angeliki Margoni, Athanasios G. Papavassiliou

**Affiliations:** 1 First University Department of Respiratory Medicine, ‘Sotiria’ Chest Hospital, Medical School, National and Kapodistrian University of Athens, 11527 Athens, Greece; 2 Department of Biological Chemistry, Medical School, National and Kapodistrian University of Athens, 11527 Athens, Greece; amaliasof@med.uoa.gr (A.A.S.); mmargoni@med.uoa.gr (A.M.)

**Keywords:** precision oncology, artificial intelligence, machine learning, deep learning, immunoengineering, drug discovery

## Abstract

Artificial intelligence (AI) is reshaping molecular oncology by making complex biological data tractable and by extending the reach of precision drug discovery. Its emerging value is particularly evident at the interface of immunoengineering and targeted therapy, where computational models can help prioritize tumor-selective antigens, predict neoantigen immunogenicity, optimize engineered receptors, support antibody and antibody–drug conjugate design, guide small-molecule discovery, refine genome-editing strategies, and anticipate treatment response. Across these applications, the central promise is not automation for its own sake, but a wider therapeutic window in which tumor control is increased while off-target toxicity is reduced. Yet the translational gap remains substantial. Many models are still preclinical, external validation is limited, and clinical implementation remains uncommon. This Commentary follows representative methods across a therapeutic continuum that now extends from target discovery and therapeutic engineering to imaging, liquid biopsy, resistance forecasting, combination selection, and drug repurposing. We argue that clinical value will depend on closed-loop workflows in which multimodal predictions are experimentally validated, externally tested, and longitudinally updated to guide the next therapeutic decision.

## 1. Introduction

Modern oncology is entering a phase in which therapeutic innovation increasingly depends on the ability to connect biological complexity with rational design. The challenge is no longer simply to discover more molecular alterations, but to determine which signals matter, how they can be translated into selective interventions, and how uncertainty can be managed across an expanding therapeutic landscape. This shift is transforming the relationship between experimentation, computation, and clinical judgment. Rather than operating as separate domains, they are becoming interdependent components of a continuous discovery-to-treatment cycle. Within this evolving framework, artificial intelligence (AI) is best understood as part of a broader methodological transition toward more integrated, predictive, and adaptive cancer therapeutics.

Cancer care has moved decisively beyond a one-size-fits-all model, driven by the recognition that tumors sharing the same histologic diagnosis may differ profoundly in their molecular dependencies, immune context, and therapeutic vulnerabilities. This intra- and intertumoral heterogeneity provides the biological foundation of precision oncology, in which molecular features are identified and therapeutically exploited to match the right intervention to the right disease context [1]. AI has emerged as a powerful means of interrogating this complexity because it can integrate high-dimensional biological information at a scale that is difficult to approach with conventional analytical methods alone [2]. Its relevance, however, extends beyond faster data processing. The more consequential opportunity is to use machine learning (ML) and related computational approaches to improve how therapeutic targets are selected, how molecular and cellular agents are engineered, and how treatment response is anticipated. In this sense, AI is beginning to function as a connective layer across precision oncology, linking molecular discovery with therapeutic design and, potentially, with clinical decision-making. Across the examples considered here, the computational toolbox ranges from Bayesian and ensemble learning to convolutional and graph neural networks, optimal transport, transformers, and generative models, each matched to a distinct biological or therapeutic question [2].

Perhaps the clearest expression of this principle lies in target selection, where an early computational decision can determine the safety and efficacy ceiling of everything that follows. ML can integrate transcriptomic and proteomic information to identify antigens or combinations of antigens that are enriched in malignant cells while being minimally represented in normal tissues. BayesTS, for example, applies Bayesian probabilistic modeling to RNA and protein profiles from more than 3500 normal tissues to quantify tumor specificity and flag candidate targets with a higher likelihood of off-tumor toxicity [3]. ImmunoTar similarly integrates user-provided RNA-sequencing or proteomic data with multiple public databases to generate prioritization scores for cell-surface targets relevant to chimeric antigen receptor T-cell and natural killer-cell therapies as well as antibody–drug conjugates [4]. The same logic extends to neoantigens. NeoTImmuML uses a weighted ensemble ML strategy to predict neoantigen immunogenicity [5], addressing a particularly attractive class of tumor-specific antigens created by acquired genomic alterations and absent from normal cells [6]. Computational pipelines can further support variant calling, human leukocyte antigen typing, peptide–major histocompatibility complex binding prediction, and immunogenicity assessment [7]. By improving target selection upstream, AI may help prevent costly downstream engineering around biologically unsuitable targets and thereby strengthen the foundation of personalized vaccines and other cancer immunotherapies.

Target identification alone, however, does not resolve the persistent limitations of cell-based immunotherapy, which include antigen escape, treatment resistance, insufficient persistence, functional dysfunction, and toxicity [8]. Here, AI can move from selecting the target to shaping the therapeutic receptor itself. Computational optimization of single-chain variable fragments has produced anti-CD19 receptor designs with robust predicted binding to both wild-type and variant CD19 while maintaining low predicted off-target interactions [9]. The CARMSeD model provides another example by identifying chimeric antigen receptor (CAR) constructs that are prone to self-activation and dysfunction, an approach that informed the development of bispecific CD20/CD19 CAR-T cells with improved persistence and reduced off-tumor toxicity [10]. A broader framework based on efficacy, safety, and accessibility has also been proposed for the design of next-generation smart CAR-T cells, in which AND, OR, and NOT logic gates and conditional activation systems can refine how engineered cells interpret tumor-associated cues [11]. Synthetic Notch (synNotch) receptors exemplify this concept because recognition of one tumor antigen can induce expression of a CAR directed against a second antigen, creating a programmable sequence of sensing and killing [12]. AI may also help anticipate cellular behavior before every construct is experimentally tested. The CAROT framework uses conditional optimal transport to predict heterogeneous single-cell gene-expression responses across CAR variants [13]. Collectively, these approaches suggest a transition from static receptor engineering toward increasingly programmable therapeutics whose behavior can be modeled before it is fully built.

Antibody-based therapeutics illustrate the same transition from empirical optimization toward computation-guided design. Deep learning (DL) methods can support antibody sequence and structure generation, model antibody–antigen interactions, and assist affinity maturation, thereby expanding the design space available for biotherapeutic development [14]. For antibody–drug conjugates (ADCs), AI can contribute across several interconnected stages, including target identification, linker and payload selection, pharmacokinetic optimization, patient stratification, and response prediction [15]. An integrated in silico analysis of transcriptomic, proteomic, immunohistochemical, and cell-surface datasets identified 82 candidate ADC targets and 290 target–indication combinations with improved tumor selectivity, while also assembling a data-mined catalog of potential payloads [16]. At the chemistry and manufacturing interface, an XGBoost-based model has predicted drug-to-antibody ratio during ADC synthesis with high accuracy [17]. Generative modeling is beginning to address linker design as well. Linker-GPT, a transformer-based DL framework using self-attention and reinforcement learning, generated ADC linkers with reported validity of 0.894 and novelty of 0.997, with 98.7% of generated structures meeting the study’s drug-likeness criteria [18]. The broader implication is that AI may eventually coordinate design variables that have traditionally been optimized in isolation, allowing target biology, antibody properties, linker chemistry, payload behavior, and patient selection to be considered within a more unified therapeutic framework.

A similar logic applies to small-molecule drug discovery, where AI can accelerate virtual screening, molecular docking, de novo molecular generation, and toxicity prediction in the search for more selective anticancer agents [19]. The potential value is especially clear for compounds with complex or pleiotropic biology. FK228, also known as depsipeptide, is a potent histone deacetylase inhibitor with multiple antitumor effects that include growth inhibition, induction of differentiation and apoptosis, and suppression of angiogenesis [20]. For such agents, computational approaches may help identify non-histone targets and context-dependent molecular interactions that could refine patient selection and reduce unwanted effects, even when AI was not involved in the original discovery of the drug. Generative chemical language models provide a more direct example of AI-enabled drug creation. In practical terms, these models learn the syntax of molecular representations such as SMILES strings in a manner analogous to language models learning relationships between words and then generate previously unseen chemical structures that satisfy specified physicochemical or target-related constraints. One such approach produced novel phosphoinositide 3-kinase gamma ligands with nanomolar activity that subsequently showed repression of the phosphoinositide 3-kinase and protein kinase B signaling pathway in a medulloblastoma cell model [21]. The translational boundary is also beginning to move into clinical development. Imneskibart, an interleukin-2 monoclonal antibody described as the first computationally designed monoclonal antibody to enter human trials, is being investigated in advanced solid tumors and has shown encouraging phase II signals in melanoma with a manageable safety profile [22]. These examples illustrate a continuum in which AI can contribute not only to finding a molecule, but also to understanding where and in whom that molecule may be most useful.

Genome editing represents another area in which computational guidance could meaningfully expand the precision of therapeutic engineering. AI is increasingly being integrated with clustered regularly interspaced short palindromic repeats and CRISPR-associated protein 9 systems to improve guide design, editing efficiency, and specificity while reducing off-target activity. Deep-learning tools such as DeepCRISPR, DeepHF, and CRISPRon use high-throughput sequence and epigenetic information to predict on-target performance and, in some cases, off-target propensity across different Cas9 configurations [23]. At the same time, base-editing and prime-editing platforms are broadening the genome-engineering repertoire by enabling precise sequence changes without the conventional double-strand breaks associated with standard CRISPR-Cas9 editing. These next-generation approaches are being actively explored for T-cell immunotherapies because they may reduce undesirable DNA modifications and genotoxicity while allowing more sophisticated engineering of therapeutic cells [24]. The important convergence is that increasingly precise editing technologies are arriving at the same moment that increasingly capable predictive models can help determine what to edit, where to edit, and which engineered phenotype is most likely to be therapeutically favorable.

The role of AI does not end once a therapeutic agent has been designed. It may also help determine which tumors are most likely to respond, an equally important component of precision oncology. Cancer Drug Response profile scan, or CDRscan, is an early DL model that predicted anticancer drug response by combining genomic profiles from 787 human cancer cell lines with structural information from 244 drugs [25]. More recent architectures attempt to capture greater biological complexity. DeepInsight-3D transforms high-dimensional multi-omics measurements into image-like representations that can be analyzed using convolutional neural networks, enabling drug-response prediction from data that can overwhelm more conventional analytical approaches [26]. An explainable AI framework has likewise integrated genomic, transcriptomic, and proteomic features with chemical drug descriptors to identify determinants of sensitivity and resistance across cancer cell lines [27]. These developments are promising, but they also expose a crucial distinction between computational performance and clinical utility. Predicting drug sensitivity in a curated experimental dataset is not equivalent to predicting benefit in a patient whose tumor evolves under treatment pressure, interacts with a complex microenvironment, and is influenced by comorbidity, prior therapy, and pharmacologic constraints. The clinically meaningful test will therefore be whether such models retain accuracy, interpretability, and added value when they move beyond retrospective datasets into independent validation and prospective therapeutic decision-making.

Imaging and tissue morphology provide another route by which AI can move response assessment beyond conventional size-based criteria. Deep learning can extract spatially resolved phenotypes from routine histopathology, while radiomics can quantify intratumoral architecture and temporal change from serial imaging. ENLIGHT-DeepPT, for example, first imputes tumor transcriptomic profiles from hematoxylin-and-eosin-stained slides and then uses the inferred expression state to predict response to targeted and immune therapies across multiple cancer types [28]. In clear-cell renal cell carcinoma, an interpretable histopathology-based deep-learning model reconstructed an angiogenesis score and predicted response to anti-angiogenic therapy in both real-world and IMmotion150 cohorts [29]. At the radiological level, a multicenter habitat-based computed tomography radiomics model captured spatial–temporal heterogeneity in resectable non-small cell lung cancer and predicted major pathological response to neoadjuvant chemoimmunotherapy, with an area under the receiver operating characteristic curve of up to 0.85 in external validation [30]. More recently, Path2Space inferred spatial gene-expression patterns directly from breast-cancer histology and identified spatial biomarkers associated with therapeutic response, illustrating how AI may connect tissue architecture with molecular state at a scale not feasible with routine spatial-transcriptomic profiling [31]. These approaches could accelerate treatment assessment by extracting actionable information from data already generated during routine care, although prospective evidence is still required to show that earlier or more granular predictions improve patient outcomes.

Liquid biopsy extends this logic into a longitudinal dimension. Because circulating tumor DNA can be sampled repeatedly, machine-learning models can combine baseline genomic features and on-treatment molecular dynamics to support rapid, minimally invasive treatment assessment. In advanced non-small cell lung cancer, a support vector machine trained on pretreatment circulating tumor DNA whole-exome sequencing from the OAK cohort and validated in the POPLAR cohort and an independent multicenter cohort was developed to predict durable clinical benefit from immune checkpoint inhibition [32]. Complementary single-cell approaches can interrogate resistance at the level of subclonal states. PERCEPTION uses single-cell tumor transcriptomics to model drug sensitivity and successfully stratified response in multiple myeloma and breast cancer while capturing the emergence of resistance in lung cancer treated with tyrosine kinase inhibitors [33]. Together, these strategies point toward adaptive precision oncology in which therapeutic choices are not fixed at baseline but are reconsidered as molecular evidence of response or resistance emerges.

AI may also reduce two search-space problems that are central to precision therapeutics, namely the selection of rational drug combinations and the identification of new uses for existing agents. In pancreatic cancer, machine-learning approaches including graph convolutional networks and random forests were used to predict synergy across approximately 1.6 million candidate drug combinations, with subsequent experimental testing confirming multiple synergistic pairs [34]. Drug repurposing offers a parallel opportunity because compounds with established pharmacological and safety information can be computationally matched to newly recognized disease dependencies. TxGNN, although developed across a broad disease spectrum rather than specifically for oncology, illustrates this strategy by combining a biomedical knowledge graph, graph neural networks, and metric learning to generate interpretable drug-repurposing predictions even for diseases with limited existing therapeutic data [35]. In precision oncology, analogous approaches could connect tumor genotype or pathway state with approved or clinically characterized agents, potentially shortening the path from molecular observation to a testable therapeutic hypothesis. Such predictions should nevertheless be regarded as prioritization tools and require tumor-specific functional and prospective clinical validation.

Taken together, these developments motivate a closed-loop multimodal framework for AI-enabled precision oncology. Rather than treating omics, pathology, imaging, liquid biopsy, and clinical variables as isolated data streams, these inputs could be integrated by interpretable models that generate ranked therapeutic hypotheses. The output may be a target, engineered receptor, drug, combination, repurposing opportunity, or resistance mechanism, but each prediction should re-enter experimental and clinical validation before it influences care. Treatment-generated data would then update the patient state and refine the next decision, shifting AI from one-time prediction toward iterative hypothesis testing (Figure 1). Making such a framework credible will require harmonized multi-institutional datasets, predefined external validation, explicit uncertainty estimates, transparent reporting, and prospective studies that test whether AI changes management compared with current standards. The objective is therefore not maximal algorithmic complexity, but a learning system in which biological plausibility, reproducibility, and clinical utility constrain every computational step.

The translational gap remains the central challenge for the field. Despite the rapid expansion of AI applications across cancer therapeutics, most remain preclinical or at an early stage of clinical implementation. A systematic review and meta-analysis of 158 DL studies focused on cancer treatment prediction found substantial methodological inconsistency, frequent risk of bias, limited external validation, and clinical implementation in only 9% of the evaluated models [36]. Such findings matter because precision oncology can be undermined by a false sense of precision. A model may be highly accurate within its development dataset yet fail to generalize across institutions, populations, assay platforms, or evolving standards of care. Human oversight therefore remains indispensable, not because computational systems lack value, but because therapeutic decisions require biological interpretation, clinical context, assessment of uncertainty, and judgment about trade-offs that are not captured by a performance metric alone. The parallel proliferation of AI tools also makes standardized benchmarking, transparent reporting, external validation, and reproducible development frameworks increasingly important. In engineered therapies, these computational standards must ultimately connect with equally rigorous experimental and manufacturing validation if an in silico advantage is to become a clinically meaningful one.

## 2. Conclusions

AI is therefore best viewed not as an autonomous replacement for oncologists, cancer researchers, or established translational pipelines, but as an enabling layer that can make each of them more precise. Its current applications already span tumor-antigen and neoantigen discovery, receptor and binding-domain optimization, prediction of cellular function and toxicity, antibody and ADC design, small-molecule discovery, genome editing, and drug-response modeling. By reducing the number of candidate targets or therapies that require experimental screening and by extracting predictive signals from routinely generated data, these approaches could shorten the time to a therapeutic decision. Whether that efficiency improves survival, quality of life, or treatment-related toxicity remains a prospective clinical question rather than an assumed consequence of model performance. Across these settings, the unifying objective is to widen the therapeutic window by increasing tumor selectivity while reducing injury to healthy tissues. The next phase of progress will depend less on producing ever more sophisticated algorithms than on building closed loops in which computational predictions are biologically interpretable, experimentally tested, externally validated, and clinically actionable. The decisive question for precision oncology is therefore not whether AI can generate compelling predictions, but whether those predictions can survive biological complexity and improve real therapeutic decisions. If that standard is met, AI will not replace the experimental and clinical foundations of oncology. It will help make them more exact, more efficient, and more capable of revealing therapeutic opportunities that might otherwise remain hidden.

## Figures and Tables

**Figure 1:**
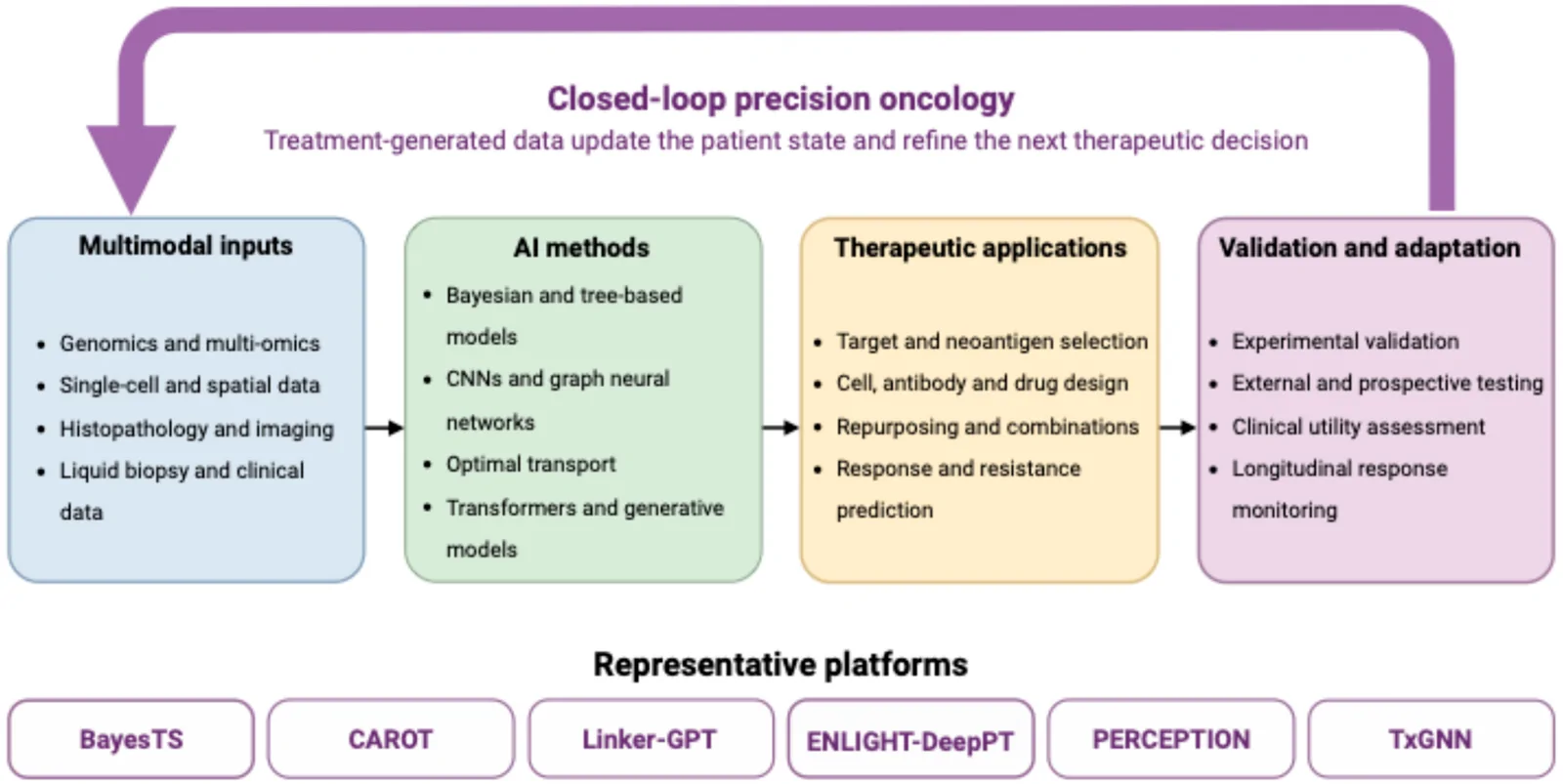
From fragmented AI applications to a closed-loop precision-oncology framework. Multimodal inputs are analyzed using complementary computational approaches and translated into therapeutic applications spanning target selection, engineered therapies, drug design, repurposing, combinations, and response or resistance prediction. Representative platforms discussed in the text are shown below the workflow. Experimental, external, and prospective validation form a feedback loop in which treatment-generated data update the patient state and refine subsequent therapeutic decisions. AI, artificial intelligence; CNNs, convolutional neural networks; BayesTS, Bayesian Tumor Specificity; CAROT, chimeric antigen receptor optimal transport; GPT, generative pre-trained transformer; ENLIGHT-DeepPT, ENLIGHT coupled with Deep Pathology for Transcriptomics; PERCEPTION, PERsonalized Single-Cell Expression-Based Planning for Treatments In Oncology. TxGNN, Therapeutic Graph Neural Network. Created in BioRender. Papavassiliou, K. (2026) https://BioRender.com/z6u431n (access on 18 September 2026).

## Data Availability

No new data were created or analyzed in this study. Data sharing is not applicable to this article.
